# Safety, outcomes, and pharmacokinetics of isavuconazole as a treatment for invasive fungal diseases in pediatric patients: a non-comparative phase 2 trial

**DOI:** 10.1128/aac.00484-24

**Published:** 2024-11-14

**Authors:** Heidi Segers, Jaime G. Deville, William J. Muller, Angela Manzanares, Amit Desai, Michael Neely, Victoria Bordon, Benjamin Hanisch, Alvaro Lassaletta, Brian T. Fisher, Julie Autmizguine, Andreas H. Groll, Shamim Sinnar, Rodney Croos-Dabrera, Marc Engelhardt, Mark Jones, Laura L. Kovanda, Antonio C. Arrieta

**Affiliations:** 1 Department of Pediatric Hemato-Oncology, University Hospital Leuven, Leuven, Belgium; 2 Department of Oncology – Pediatric Oncology, KU Leuven, Leuven, Belgium; 3 Department of Pediatrics, University of California, Los Angeles, California, USA; 4 Ann & Robert H. Lurie Children’s Hospital of Chicago, Chicago, Illinois, USA; 5 Pediatric Infectious Disease Unit, Hospital 12 de Octubre, Madrid, Spain; 6 Astellas Pharma Global Development, Inc., Northbrook, Illinois, USA; 7 University of Southern California Children's Hospital Los Angeles, Los Angeles, California, USA; 8 Ghent University Hospital, Ghent, Belgium; 9 Children's National Hospital, Washington, DC, USA; 10 Hospital Infantil Universitario Niño Jesús, Madrid, Spain; 11 Children's Hospital of Philadelphia and The Perelman School of Medicine at The University of Pennsylvania, Philadelphia, USA; 12 Department of Pharmacology and Physiology, Université de Montréal, Montréal, Canada; 13 Research Center, CHU Sainte-Justine, Montréal, Canada; 14 Infectious Disease Research Program, Center for Bone Marrow Transplantation and Department of Pediatric Hematology/Oncology, Children’s University Hospital Münster, Münster, Germany; 15 Basilea Pharmaceutica International Ltd., Allschwil, Switzerland; 16 Children’s Hospital of Orange County, Orange, and University of California, Irvine, California, USA; University of Iowa, Iowa City, Iowa, USA

**Keywords:** isavuconazole, invasive fungal diseases, pediatrics

## Abstract

Invasive aspergillosis (IA) and mucormycosis (IM) cause significant morbidity and mortality in immunocompromised and/or hospitalized patients. Isavuconazonium sulfate, a prodrug of the antifungal triazole isavuconazole, has been approved for treatment of IA and IM in adults; and was recently approved in children. This study describes the outcomes, safety, and pharmacokinetics of isavuconazole for the treatment of proven, probable, or possible IA or IM in children. In this phase 2, open-label, non-comparative study, patients aged 1 to <18 years with at least possible invasive mold disease were enrolled across 10 centers in the US, Spain, and Belgium from 2019 to 2022. Patients received 10 mg/kg isavuconazonium sulfate daily (maximum 372 mg; equivalent to 5.4 mg/kg or 200 mg isavuconazole) for up to 84 (IA) or 180 days (IM). Outcomes included rates of all-cause case fatality, overall response, treatment-emergent adverse events (TEAEs), and pharmacokinetics. Of 31 patients enrolled, 61.3% were 1–<12 years old; 58.1% had underlying hematologic malignancies. The successful overall response rate at the end of treatment was 54.8%. Day 42 all-cause case fatality was 6.5%; 93.5% experienced TEAEs, and two patients discontinued treatment due to drug-related TEAEs. Dosing at 10 mg/kg (maximum dose: 372 mg) met the pre-defined exposure threshold of above the 25th percentile for the area under the concentration-time curve (≥60 mg·h/L). Simulated doses of 15 mg/kg improved drug exposures in patients aged 1–<3 years. Isavuconazole was well tolerated in children, with exposure consistent with adult studies. Successful response was documented in 54.8% of patients.

**CLINICAL TRIALS:**

This study is registered at ClinicalTrials.gov as NCT03816176.

## INTRODUCTION

Invasive aspergillosis (IA) and mucormycosis (IM) are life-threatening invasive fungal diseases (IFDs) associated with significant morbidity and case fatality in immune-compromised children (1, 2).

Currently, recommended therapies for IA and IM include voriconazole (IA: primary treatment) and liposomal amphotericin B (IM: primary treatment), with newly developed mold-active triazoles as alternatives according to the Infectious Diseases Society of America and the European Confederation of Medical Mycology guidelines (3–5). Isavuconazole is a triazole approved in the US for the treatment of IA or IM in adults and children aged 1 year and older, as well as in Europe for the treatment of adults with IA or adults with IM for whom amphotericin B is inappropriate. Isavuconazole is administered as the prodrug isavuconazonium sulfate (6–8). In adults, the clinical dosing regimen (prodrug doses of 372 mg/day, equivalent to 200 mg/day isavuconazole) achieved exposures adequate to treat infections in phase 3 double-blind, global, multicenter, comparative (SECURE) study of adults with proven, probable, or possible *Aspergillus* and/or other filamentous fungi (9); isavuconazole was non-inferior to voriconazole in day 42 all-cause case fatality (10). Furthermore, in a global single-arm study (VITAL) in adults with IM, isavuconazole showed similar clinical response outcomes and all-cause case fatality to that of amphotericin B-based treatments in previous trials of primary or salvage therapy, as well as a matched case-controlled comparison (11).

Recently, an open-label phase 1 study conducted in the US evaluated the pharmacokinetics and safety of isavuconazole in immunocompromised pediatric patients (from 1 to <18 years of age) at risk of IFDs and reported that isavuconazole was generally well tolerated, with a safety profile and study drug exposures comparable to those in a previous study in adult patients (12). The aim of this phase 2 study was to further investigate the outcomes, safety, and pharmacokinetics, including dosing recommendations of isavuconazole (administered as the prodrug isavuconazonium sulfate) for the treatment of proven, probable, or possible IA or IM in a pediatric population.

## MATERIALS AND METHODS

### Study design

This was a phase 2, open-label, non-comparative, multicenter study to investigate the safety, outcomes, and pharmacokinetics of isavuconazole (administered as isavuconazonium sulfate) in pediatric patients for the treatment of IA or IM (NCT03816176). All patients were enrolled with a diagnosis of proven/probable IA or IM, or possible IFD per European Organization for Research and Treatment of Cancer/Mycoses Study Group (EORTC/MSG) 2008 criteria (13), required systemic antifungal therapy, and were screened as eligible within 5 days prior to treatment initiation. Patients enrolled with a diagnosis of possible IFD had to receive appropriate testing within 10 days to assess for proven/probable IA or IM. If the test results did not meet the criteria for proven/probable IA or IM, they could remain in the study under the category of possible IFD, as per the inclusion criteria.

**Fig 1:**
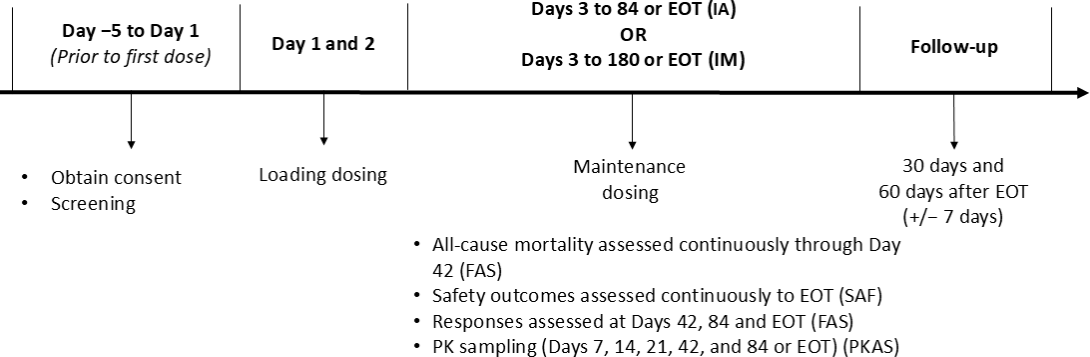
Study design. All patients were assigned to open-label treatment via IV or oral route at the discretion of the investigator. FAS, full analysis set; EOT, end of treatment; IA, invasive aspergillus; IM, invasive mucormycosis; IV, intravenous; PKAS, pharmacokinetic analysis set; SAF, safety analysis set.

Eligible patients were enrolled in 10 centers in the US, Spain, and Belgium from 2019 to 2022, and assigned to open-label intravenous (IV) or oral isavuconazonium sulfate. Patients aged between 1 and <18 years old could receive study drugs intravenously. Oral dosing was only available to patients 6 to <18 years of age with a body weight of ≥12 kg who were able to swallow capsules. When clinically appropriate, patients received treatment until a successful outcome was achieved, as judged by an investigator (Supplemental methods), or up to a maximum duration of therapy was reached (84 days for IA or 180 days for IM), whichever came first, with follow-up post-treatment for up to 60 days (Fig. 1). If testing suggested IFD of other than proven or probable IA or IM, treatment was switched to an antifungal more specific/effective for the pathogen.

Treatment could be stopped at the investigator’s discretion if it was determined that continuing would not be in the patient’s best interest, including due to adverse events.

### Patients

The study population consisted of pediatric patients (1 to <18 years of age) who met eligibility criteria, including proven/probable IA or IM, or possible IFD, per the EORTC/MSG 2008 criteria (13).

For patients with underlying hematologic malignancy or recipients of hematopoietic cell transplants with clinical and radiologic features consistent with IFD, the following galactomannan levels were sufficient to satisfy the EORTC/MSG 2008 mycological criteria (13) to define probable IA: a single value galactomannan level of ≥1.0 for serum or bronchoalveolar lavage fluid, or two serum galactomannan levels of ≥0.5 from two separate samples.

Patients were excluded if they had been diagnosed with an IFD other than proven, probable, or possible IA or IM at the time of enrollment; had chronic aspergillosis, aspergilloma, or allergic bronchopulmonary aspergillosis; were treated with mold-active systemic antifungal therapy effective against the primary IFD for >4 days during the 7 days preceding the first dose of the study drug; had evidence of hepatic dysfunction; or were unlikely to survive for ≥30 days in the investigator’s opinion. Prior use of prophylactic antifungal therapy was acceptable; however, in case of breakthrough IA while on prophylactic mold-active azole drugs, additional approval was required by the study medical monitor.

### Treatments and procedures

Patients received a loading regimen of 10 mg/kg isavuconazonium sulfate (equivalent to 5.4 mg/kg isavuconazole), up to a maximum of 372 mg of isavuconazonium sulfate (equivalent to 200 mg isavuconazole) every 8 h (±2 h) for six total doses over days 1 and 2 (Fig. 1). A once-daily maintenance dose of 10 mg/kg isavuconazonium sulfate (up to a maximum of 372 mg) was then administered for up to 84 days (IA) or 180 days (IM), or until a successful response was achieved (defined in Supplemental methods), whichever occurred first (Fig. 1). Maintenance treatment was started 12 to 24 h after administration of the last loading dose; subsequent maintenance doses were administered once daily. Details on dose by patient body weight are included in Table S1 and Supplemental methods.

IV treatment was administered as an isavuconazonium sulfate infusion; oral treatment was administered as isavuconazonium sulfate 74.5 mg capsules (equivalent to 40 mg isavuconazole). Dose selection was informed by data from the preceding pediatric phase 1 study (12). Doses for the two administration routes (oral and IV) were equivalent on a mg:mg basis (Table S1). The route of administration could be changed after study initiation, as determined by the investigator and there was no need to repeat the loading dose if the change was made after day 2. Concomitant use of other systemic antifungal therapy was prohibited during the study period, though concomitant antifungal therapy that ended on the date of study drug initiation or occurred during follow-up after the end of treatment (EOT) was permitted.

### Outcomes

The primary clinical outcome of this study was an all-cause case fatality rate through day 42. The primary safety outcome was treatment-emergent adverse events (TEAEs). TEAEs were identified by continuous recording of adverse events (AEs) as well as via systematic monitoring of vital signs, results of electrocardiograms (ECGs), and specified laboratory results at predetermined intervals (Supplemental methods) during and until EOT. Laboratory parameters and other clinical evaluations were also assessed to determine any occurrence of Hy’s Law (alanine aminotransferase [ALT] and/or aspartate aminotransferase [AST] >3× upper limit of normal [ULN], alkaline phosphatase <2× ULN, and total bilirubin >2× ULN, in the absence of another etiology causing the biochemical abnormalities).

Secondary outcomes included all-cause case fatality rate through day 84 and EOT; additionally, the overall response was assessed by an independent adjudication committee (comprised of global pediatric infectious disease experts) based on prespecified success criteria (defined in Supplemental methods) through day 42, day 84, and EOT. Pharmacokinetic (PK) outcomes of isavuconazole were also assessed as detailed below.

### Statistical analyses

No formal sample size calculation was performed in this study; however, a sample size of approximately 30 patients was planned, including at least five evaluable patients for each age cohort of 1 to <12 years and ≥12 to <18 years. As an open-label trial without a comparator group, all analyses were descriptive with no formal inferential analyses performed. In general, continuous data were summarized by number of patients, mean, standard deviation (SD), minimum, median, and maximum; categorical data were summarized by frequency and percentage.

Clinical outcomes were assessed in the full analysis set (FAS), which consisted of all patients who received ≥1 dose of the study drug. Safety outcomes were assessed in the safety analysis set (SAF), which was identical to the FAS. Patients with possible IA or IM who were subsequently diagnosed with probable or proven IFD other than IA or IM were not excluded from the FAS or SAF.

For all outcomes, IFDs were categorized into the following subgroups: proven or probable IA; proven or probable IM; possible IFD; and other IFD (excluding IA or IM).

### Pharmacokinetic analysis

PK outcomes of isavuconazole included plasma trough concentrations on days 7, 14, 21, 42, and 84, and EOT. In addition, 24-h PK samples were obtained on any day between days 14 and 42 while the patient was still receiving the study drug. Samples for 24-h PK during IV administration were as follows: within 1 h prior to the next infusion; immediately following infusion; within 4 to 10 h; and within 16 to 24 h after the start of the infusion. Samples for 24-h PK during oral administration were as follows: within 1 h prior to the next oral dose; at 1, 3, and 4 h post-dose; within 6 to 8 h post-dose; at 24 h post-dose.

All patients who received ≥1 dose of the study drug and who had at least one blood plasma concentration available were included in the population pharmacokinetic analysis (PPK).

Plasma PK concentrations from 28 patients in the current study were added to the previously established PPK model in a similar population and analyzed (12). All plasma samples collected from these patients were included in this analysis data set and AUC values for pediatric participants were derived using their individual empirical Bayes parameter estimates. The PPK model consisted of three compartments with combined zero and first-order absorption and linear elimination. The model also included allometric scaling based on body weight to scale size-related changes in clearance and volume of distribution. None of the covariates (liver function tests, age, sex, race, and ethnicity) tested in the model were considered to be statistically significant.

The area under the concentration-time curve at steady state (AUC_ss_) was derived for all subjects based on the estimated clearance values and dose for each individual, and group curves were established for three age cohorts (1 to <6 years, ≥6 to <12 years, and ≥12 to <18 years).

### Pediatric dose confirmation

Drug exposure values (AUC_ss_) from each age group were evaluated against a predefined target exposure range for the pediatric population to confirm the adequacy of the recommended clinical dosing regimen in children aged 1 to <18 years. The lower threshold value of the target AUC range was derived from the 25th percentile (60 mg∙h/L) of the drug exposure range in the phase 3 SECURE clinical study of adult patients receiving the recommended clinical dose (9), and the upper threshold value (233 mg∙h/L) was derived from the lower end of the drug exposures range from a previous phase 1 high-dose clinical study where toxicity was experienced (14). Since formal exposure-response analysis in the adult population from the SECURE study did not determine a plasma drug exposure associated with treatment success, the lower bound of the target plasma drug exposure range for pediatric patients was arbitrarily set at the 25th percentile value for the AUC from the phase 3 SECURE study (9), as described in the preceding pediatric phase 1 study (12).

When the drug exposure was not considered adequate within a particular age group, individual patient simulations (using empirical Bayes estimates) and Monte Carlo simulations (MCS) (using mean population values) were performed with different dosing regimens. For the MCS, a total of 800 patients were simulated and AUC_ss_ were estimated.

The detailed PPK modeling analysis will be published elsewhere.

## RESULTS

### Patient demographics and characteristics

Overall, 31 patients were enrolled in the study and included in the FAS and SAF (Table 1; Table S2), of whom 28 had at least one blood plasma concentration available for PK analyses.

**TABLE 1:** Patient baseline characteristics (FAS)^*a*^

Characteristic	Subgroup				
	Proven/probable IA (*n* = 12)	Proven/probable IM (*n* = 1)	Possible IFD^*b*^*^,^*^*c*^ (*n* = 16)	Other IFD^*d*^ (*n* = 2)	Total (*N* = 31)
Age: median (range), y	10.0 (1–16)	14.0 (14–14)	9.0 (1–17)	12.0 (11–13)	10.0 (1–17)
1–<12 y, n (%)	7 (58.3)	0	11 (68.8)	1 (50.0)	19 (61.3)
≥12–<18 y, n (%)	5 (41.7)	1 (100)	5 (31.3)	1 (50.0)	12 (38.7)
Ethnicity: non-Hispanic/Latino, n (%)	7 (58.3)	0	12 (75.0)	0	19 (61.3)
Female, n (%)	10 (83.3)	0	13 (81.3)	2 (100)	25 (80.6)
Race, n (%)					
White	4 (33.3)	1 (100)	13 (81.3)	1 (50.0)	19 (61.3)
Black or African American	0	0	1 (6.3)	0	1 (3.2)
Asian	4 (33.3)	0	1 (6.3)	0	5 (16.1)
American Indian or Alaska Native	0	0	0	0	0
Native Hawaiian or other Pacific Islander	0	0	0	0	0
Other (included “unknown,” “unspecified,” “White/Asian,” and “Latino and/or Hispanic”)	2 (16.7)	0	1 (6.3)	1 (50.0)	4 (12.9)
Not specified	2 (16.7)	0	0	0	2 (6.5)
Underlying disease/condition, n (%)					
Malignancy	6 (50.0)	1 (100)	11 (68.8)	1 (50.0)	19 (61.3)
ALL	3 (25.0)	1 (100)	5 (31.3)	0	9 (29.0)
B-LL	1 (8.3)	0	0	0	1 (3.2)
AML	1 (8.3)	0	3 (18.8)	1 (50.0)	5 (16.1)
MDS to AML	0	0	1 (6.3)	0	1 (3.2)
NHL	0	0	2 (12.5)	0	2 (6.5)
Other solid tumor^*e*^	1 (8.3)	0	0	0	1 (3.2)
Non-malignancy	6 (50.0)	0	5 (31.3)	1 (50.0)	12 (38.7)
Immune disorder	2 (16.7)	0	1 (6.3)	1 (50.0)	4 (12.9)
Other^*f*^	4 (33.3)	0	4 (25.0)	0	8 (25.8)

^*a*^ ALL, acute lymphoblastic leukemia; AML, acute myeloblastic leukemia (includes relapsed disease); B-LL, B-cell lymphoblastic leukemia/lymphoma; MDS to AML, myelodysplastic syndrome transformed to AML; NHL, non-Hodgkin lymphoma.

^*b*^ Two of the patients with possible IFD was subsequently determined by the independent adjudication committee as not meeting the EORTC/MSG 2008 criteria (13) for possible IFD.

^*c*^ An investigator assessment of IFD diagnosis was used; diagnostic tests to assess whether the disease was “proven” or “probable” IA or IM, according to the EORTC/MSG 2008 criteria (13), were completed within 10 days after the first dose of the study drug.

^*d*^ Other IFDs were defined as IFDs assessed not to be IA or IM.

^*e*^ Medulloblastoma.

^*f*^ Includes systemic lupus erythematosus, solid organ transplant, multi-visceral transplant, central venous access (congenital ileal atresia), cystic adenomatoid malformation (congenital), Fanconi’s anemia, primary pulmonary hypertension, and aplastic anemia.

At baseline, the mean (SD) age of patients was 9.7 (5.0) years, and the majority were White (61.3%, 19/31) and female (80.6%, 25/31) (Table 1). In SAF, the most commonly used prior antifungal prophylactic medications were fluconazole (32.3%, 10/31), liposomal amphotericin B (29.0%, 9/31), or micafungin (19.4%, 6/31). Ten patients started or ended systemic antifungal therapy (other than the study drug) on the same day that the study drug was initiated or ended, while two patients received systemic antifungal therapy for brief periods during treatment with the study drug from days 1 to 2 and days 69 to 71, respectively.

At enrollment, all patients met the necessary criteria for at least possible IFD as assessed by the investigator and were included in this study; however, two were eventually diagnosed with proven non-IA/IM IFD (other IFD; coccidioidomycosis and fusariosis) within 10 days after the first dose of the study drug Fig. S1.

Of the remaining 29 patients with at least possible IFD, 13 (44.8%) had proven or probable IA (12/13) or IM (1/13), and 16 (55.2%) remained as possible IFD. Across all patients, there was general agreement between the adjudication committee and the investigators, except for two of the 16 patients categorized as possible IFD by the investigator. These patients were enrolled based on positive galactomannan levels, which is one of the mycological criteria outlined in the EORTC/MSG 2008 criteria (13); however, neither patient had the EORTC/MSG-specific host factors nor met the protocol-specified diagnostic criteria for possible IFD including IA/IM. One patient had an underlying diagnosis of systemic lupus erythematosus, while the second patient had a history of multi-visceral transplants and was PCR-positive for *Aspergillus fumigatus* at enrollment.

In general, the most common underlying conditions were malignancies (61.3%, 19/31), which were predominantly acute lymphoblastic leukemia (29.0%, 9/31) and newly diagnosed or relapsed acute myeloblastic leukemia (16.1%, 5/31) (Table 1). Nearly two-thirds of patients (64.5%, 20/31) had recently resolved or ongoing neutropenia, approximately half (45.2%, 14/31) were receiving other recognized T-cell immune suppressive regimens and approximately one-third (32.3%, 10/31) had prolonged use of corticosteroids.

### Treatment

In SAF, the mean and median duration of isavuconazole treatment were 57.7 and 55.0 (range, 2–181) days, respectively, with 61.3% (19/31) receiving 42 days or more of isavuconazole treatment. At EOT, 61.3% (19/31) of patients in the SAF completed treatment without premature discontinuation of isavuconazole; 63.2% (12/19) of these patients achieved the maximum permitted treatment duration (IA, 84 days; IM, 180 days) and the remaining seven participants finished earlier upon achievement of a successful outcome (36.8%, 7/19) (Table S2 and S3). For the 12 patients who discontinued study treatment prior to completion or successful outcome, the reasons for discontinuation of treatment, based on the investigator’s categorization, were lack of clinical improvement (*n* = 4), AEs (*n* = 3), and others (*n* = 5).

### Clinical outcomes

#### All-cause case fatality

The primary clinical outcome of all-cause case fatality through day 42 was 6.5% (2/31) (FAS, Table 2; Table S4). The causes of death were related to the progression of IFD and septic shock; the duration of time between isavuconazole discontinuation and death was 5 days for the patient with progression of IFD and 20 days for the patient with septic shock. One additional death, attributed to cardiovascular collapse, occurred between days 42 and 84, for a total of three deaths occurring during follow-up (9.7%, 3/31); the duration of time between isavuconazole discontinuation and death was 23 days for this patient with cardiovascular collapse (Table 2; Table S4). All deaths occurred after treatment with the study drug had ended, and the investigator did not consider the deaths to be treatment-related.

**TABLE 2:** All-cause case fatality (FAS)^*a*^

Timepoint	Subgroup					
	Outcome	Proven/probable IA (*n* = 12)	Proven/probable IM (*n* = 1)	Possible IFD^*b*^^,*c*^ (*n* = 16)	Other IFD^*d*^ (*n* = 2)	Total (*N* = 31)
Day 42	All-cause case fatality, n (%)	1 (8.3)	0	1 (6.3)	0	2 (6.5)
	95% CI (%)	(0.21, 38.48)	(0.00, 97.50)	(0.16, 30.23)	(0.00, 84.19)	(0.79, 21.42)
	Known deaths	1 (8.3)	0	1 (6.3)	0	2 (6.5)
Day 84	All-cause case fatality, n (%)	2 (16.7)	0	1 (6.3)	0	3 (9.7)
	95% CI (%)	(2.09, 48.41)	(0.00, 97.50)	(0.16, 30.23)	(0.00, 84.19)	(2.04, 25.75)
	Known deaths	2 (16.7)	0	1 (6.3)	0	3 (9.7)
EOT	All-cause case fatality, n (%)	0	0	0	0	0
	Known deaths	0	0	0	0	0

^*a*^ CI, confidence interval.

^*b*^ Two of the patients with possible IFD were subsequently determined by the independent adjudication committee as not meeting the EORTC/MSG 2008 criteria (13) for possible IFD.

^*c*^ An investigator assessment of IFD diagnosis was used; diagnostic tests to assess whether the disease was “proven” or “probable” IA or IM, according to the EORTC/MSG 2008 criteria (13), were completed within 10 days after the first dose of the study drug.

^*d*^ Other IFDs were defined as IFDs assessed not to be IA or IM.

#### Adjudication committee-assessed overall response

**Fig 2:**
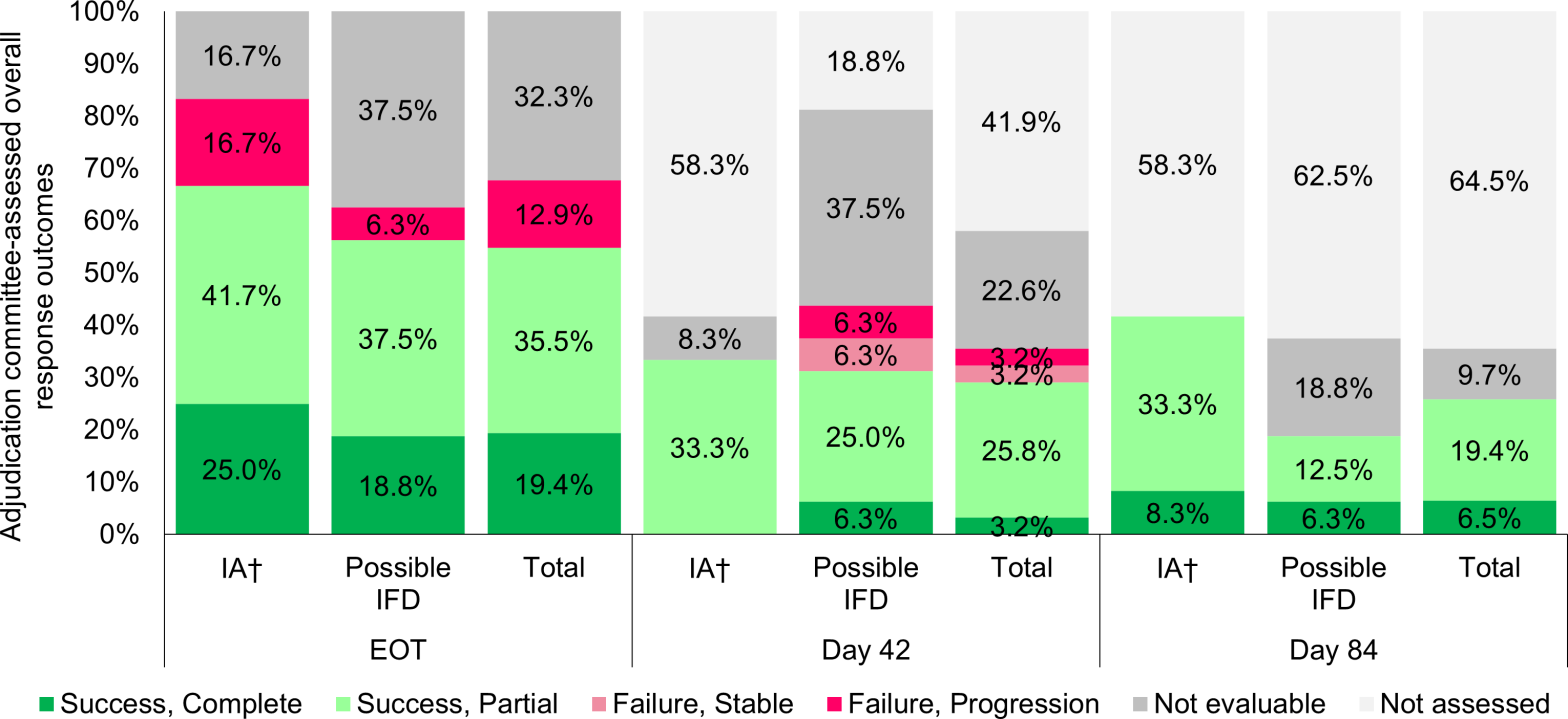
Adjudication committee-assessed overall response (FAS). AC, adjudication committee; FAS, full analysis set; IA, invasive aspergillosis; IFD: invasive fungal disease; IM, invasive mucormycosis; EOT, end of treatment. Not evaluable includes those assessments that could not be made due to lack of data and no assessment performed by the AC because the patient did not reach the assessment day (discontinued treatment prior to either day 42 or day 84 of therapy). The overall response was based on a composite of clinical, mycological, and radiological responses with success criteria assessed. At EOT, the only patient in the IM subgroup was assessed as failure (due to progression) and both patients in the other IFD subgroup were considered “not evaluable” for overall response. ^†^Proven/probable.

Across all patients in the FAS, at days 42, 84, and EOT, successful adjudication committee-assessed overall response rates were 29.0% (9/31), 25.8% (8/31), and 54.8% (17/31), respectively. Additionally, 66.7% (8/12) of patients with proven or probable IA were assessed by the adjudication committee as having a successful overall response at EOT (Fig. 2). Of note, 13/31 (41.9%) and 20/31 (64.5%) patients did not undergo adjudication committee-assessments at days 42 and 84, respectively, because their treatment had been terminated before the assessment time points.

### Safety

#### Adverse events

TEAEs (*n* = 415) were reported in 93.5% (29/31) of patients; 9.7% (3/31) of all patients with TEAEs and 6.5% (2/31) with drug-related TEAEs had events that led to withdrawal of treatment (Table 3). Through EOT, 29.0% (9/31) of patients experienced a drug-related TEAE, the most common being nausea (6.5%, 2/31) and infusion site pain (6.5%, 2/31) (Table S5). Notably, the large majority of drug-related TEAEs (*n* = 24 events) were rated as mild/moderate (95.8%, 23/24) in intensity, with one severe drug-related TEAE (hypotension) (Table S5). The two drug-related TEAEs that lead to withdrawal were hypotension (1/24, 4.2%; non-serious) and injection site reaction (1/24, 4.2%; serious); the hypotensive event was severe in intensity, occurring on day 1 of IV treatment and resulting in withdrawal by day 6. A comprehensive patient-level breakdown of drug-related adverse events by treatment duration and withdrawals is detailed in Table S3.

**TABLE 3:** Overview of TEAEs (SAF)^*a*^

Parameter	Subgroup				
	Proven/probable IA (*n* = 12)	Proven/probable IM (*n* = 1)	Possible IFD^*b*^^*,^c^*^ (*n* = 16)	Other IFD^*d*^ (*n* = 2)	Total (*N* = 31)
Overall TEAE, n (%)^*e*^	11 (91.7)	1 (100)	15 (93.8)	2 (100)	29 (93.5)
Drug-related	3 (25.0)	1 (100)	5 (31.3)	0	9 (29.0)
Serious TEAE	9 (75.0)	0	9 (56.3)	0	18 (58.1)
Drug-related	1 (8.3)	0	0	0	1 (3.2)
TEAE leading to death	2 (16.7)	0	1 (6.3)	0	3 (9.7)
Drug-related	0	0	0	0	0
TEAE leading to treatment withdrawal	2 (16.7)	0	1 (6.3)	0	3 (9.7)
Drug-related^*f*^	1 (8.3)	0	1 (6.3)	0	2 (6.5)

^*a*^ Drug-related events, as assessed by the investigator, were those with a reasonable possibility that the event may have been caused by the study drug; if any other relationship was missing, the event was also considered as drug-related.

^*b*^ Two of the patients with possible IFD were subsequently determined by the independent adjudication committee as not meeting the EORTC/MSG 2008 criteria (13) for possible IFD.

^*c*^ An investigator assessment of IFD diagnosis was used; diagnostic tests to assess whether the disease was “proven” or “probable” IA or IM, according to the EORTC/MSG 2008 (13) criteria, were completed within 10 days after the first dose of the study drug.

^*d*^ Other IFDs were defined as IFDs assessed not to be IA or IM.

^*e*^ TEAEs were defined as adverse events observed after starting the study drug 30 days after the last dose.

^*f*^ Drug-related TEAEs that lead to withdrawal were hypotension (non-serious) and injection site reaction (serious).

The most common non-serious TEAEs (reported in >5% of patients) were pyrexia (29.0%, 9/31), diarrhea (25.8%, 8/31), vomiting (22.6%, 7/31), non-cardiac chest pain (16.1%, 5/31), stomatitis (16.1%, 5/31), nausea (12.9%, 4/31), and aphthous ulcer (12.9%, 4/31) (Table S6).

In total, 18/31 patients (58.1%) experienced a treatment-emergent serious adverse event (SAE), of which the most common were septic shock (9.7%, 3/31), febrile neutropenia (6.5%, 2/31), and stomatitis (6.5%, 2/31) (Table S7). Overall, TEAEs that lead to death occurred in 3/31 (9.7%) patients (Table 3), and all such deaths occurred after EOT.

#### Laboratory parameters and other clinical evaluations

Overall, a few (6.5%, 2/31) patients had ALT/AST levels >10 times the ULN (Table 4). Three patients had concurrent values for ALT and/or AST >3× ULN and total bilirubin >2× ULN; however, for each patient, there were other significant or confounding factors (veno-occlusive disease, multi-organ dysfunction due to septic shock, and concomitant hepatic viral infection) that ruled out Hy’s law. No other clinically significant changes from baseline in laboratory parameters were observed and there were no trends in liver function abnormalities (Table 4).

**TABLE 4:** Clinical laboratory and other evaluations (SAF)^*a*^

Parameter	Subgroup				
	Proven/probable IA (*n* = 12)	Proven/probable IM (*n* = 1)	Possible IFD^*b*^^*, c*^ (*n* = 16)	Other IFD^*d*^ (*n* = 2)	Total (*N* = 31)
ALT, n/N (%)					
>3× ULN	4/12 (33.3)	0	5/16 (31.3)	0	9/31 (29.0)
>5× ULN	1/12 (8.3)	0	2/16 (12.5)	0	3/31 (9.7)
>10× ULN	0	0	0	0	0
>20× ULN	0	0	0	0	0
AST, n/N (%)					
>3× ULN	3/12 (25.0)	0	2/16 (12.5)	0	5/31 (16.1)
>5× ULN	1/12 (8.3)	0	2/16 (12.5)	0	3/31 (9.7)
>10× ULN	0	0	2/16 (12.5)	0	2/31 (6.5)
>20× ULN	0	0	2/16 (12.5)	0	2/31 (6.5)
ALT or AST, n/N (%)					
>3× ULN	4/12 (33.3)	0	6/16 (37.5)	0	10/31 (32.3)
>5× ULN	1/12 (8.3)	0	3/16 (18.8)	0	4/31 (12.9)
>10× ULN	0	0	2/16 (12.5)	0	2/31 (6.5)
>20× ULN	0	0	2/16 (12.5)	0	2/31 (6.5)
Total bilirubin, n/N (%)					
>2× ULN	1/12 (8.3)	0	3/16 (18.8)	0	4/31 (12.9)
ALP, n/N (%)					
>1.5× ULN	0	0	0	0	0
ALT and/or AST >3× ULN AND total bilirubin >2× ULN, n/N (%)^*e*^	1/12 (8.3)	0	2/16 (12.5)	0	3/31 (9.7)
ALT and/or AST >3× ULN AND ALP <2× ULN AND total bilirubin >2× ULN, n/N (%)^*e*^	1/11 (9.1)^*f*^	0	2/15 (13.3)^*f*^	0	3/29 (10.3)^*f*^

^*a*^ ALP, alkaline phosphatase; T-bil, total bilirubin.

^*b*^ Two of the patients with possible IFD were subsequently determined by the independent adjudication committee as not meeting the EORTC/MSG 2008 criteria (13) for possible IFD.

^*c*^ An investigator assessment of IFD diagnosis was used; diagnostic tests to assess whether the disease was “proven” or “probable” IA or IM, according to the EORTC/MSG 2008 criteria (13), were completed within 10 days after the first dose of the study drug.

^*d*^ Other IFDs were defined as IFDs assessed not to be IA or IM.

^*e*^ Combination of values measured within the same day or 1 day apart.

^*f*^ Patients who potentially met Hy’s Law criteria (ALT and/or AST >3× ULN, alkaline phosphatase <2× ULN, total bilirubin >2× ULN).

Lastly, the investigators did not consider any findings in vital sign measurements, ECG measurements, or other observations to be significant and related to safety in this study.

### Pharmacokinetic analysis

**Fig 3:**
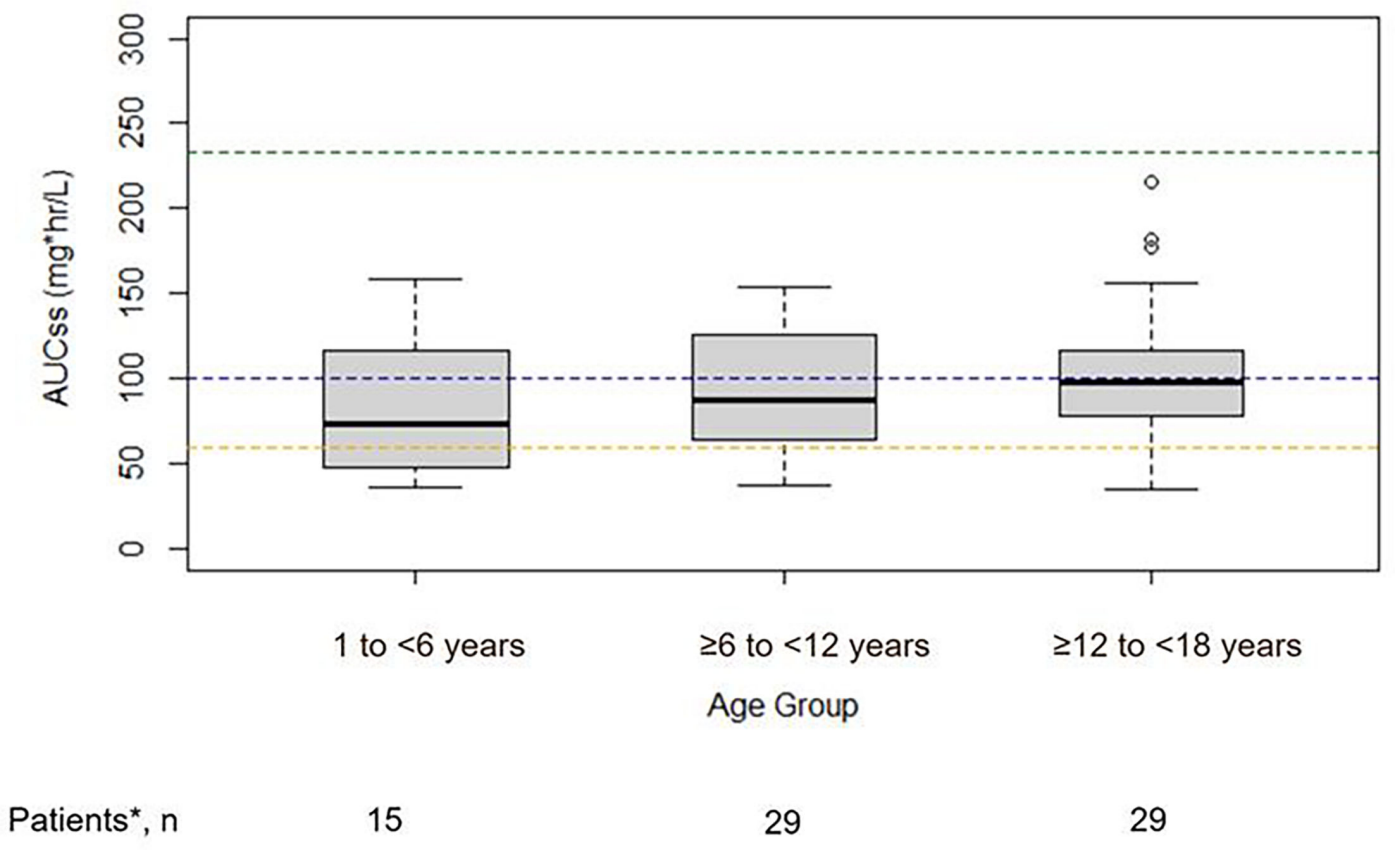
Comparison of AUC_ss_ values by age group versus previous adult studies. AUC_24_, the area under the concentration-time curve over 24 h; Boxes represent the median and 25th and 75th percentiles, whiskers represent the range of maximum and minimum values within 1.5× the interquartile range, and outliers are shown as­ circle­s. The dashed blue line is the mean AUC_ss_ (101 mg·h/L) from the SECURE study (9). The dashed green line is the minimum (233 mg·h/L) AUC_24_ value in a high-dose adult study (12, 14) (1,116 mg) with increased toxicity and represents the upper limit of the target exposure range for pediatric patients. The dashed orange line is the lower limit of the targeted exposure value (AUC_ss_: 60 mg·h/L), which is taken from the 25th percentile of observed exposures in adults from the phase 3 SECURE trial (9, 12). *Overall population based on the combined patient populations from this study and the previous phase 1 study population (12).

In addition to the 28 patients from the present study who had at least one blood plasma concentration available for PK analyses, a further 45 patients included in the previously published PK analysis were added to the current study (12). The median AUC_ss_ for all age groups was above the target exposure level (AUC_ss_: 60 mg·h/L), and 80.8% (59/73) of all patients had exposures above 60 mg·h/L (Fig. 3) (9, 12, 14). In total, 5/15 (33.3%), 6/29 (20.7%), and 3/29 (10.3%) of respective patients aged 1 to <6 years, ≥6 to <12 years, and ≥12 to <18 years had an AUC_ss_ <60 mg·h/L. Overall, no patient had an AUC_ss_ of ≥233 mg·h/L (upper threshold of target exposure range), and the median AUC_ss_ was comparable to the mean observed in adult patients from the SECURE study, across ≥6 to <12 and ≥12 to <18 years age groups (Fig. 3) (9); however, the median AUC_ss_ was lower in patients 1 to <6 years of age versus those ≥6 to <12 and ≥12 to <18 years of age. The source of the lower exposure in the less than 6-year-olds was attributed to the patients less than 3 years of age. Only 5 patients were less than 3 years of age and had derived exposure values ranging from 35.8 mg·h/L to 103.5 mg·h/L. Examination of the plasma concentration data and patient clinical data did not reveal a specific reason for the variability in this age group. Alternative dosing regimens were also simulated using the individual PK parameter estimates of each study patient aged 1 to <3 years old, as well as using mean population-based parameters estimated from the PPK analysis (MCS). These data suggest that a higher dose of 15 mg/kg isavuconazonium sulfate is warranted in patients less than 3 years of age, as it resulted in exposures above the lower end of the target exposure range in all but one patient aged <3 years old when using individual parameters, with none falling above the upper exposure target (Table 5).

**TABLE 5:** Simulated AUC_ss_ exposure values in patients aged 1 to <3 years, following 15 mg/kg doses of isavuconazonium sulfate

Age category	1 to <3 years (*n* = 5)
Mean	80.22
Median	64.27
Min-Max	53.73–155.00

## DISCUSSION

Our phase 2 study assessed the safety, outcomes, and PK of isavuconazole in pediatric patients for the treatment of proven/probable IA or IM, and possible IFD. All-cause case fatality rates were 6.5% and 9.7% through day 42 and day 84, respectively. Successful overall responses, as assessed by the independent adjudication committee, were documented in 29.0% and 54.8% of patients on day 42 and at EOT, respectively. Additionally, although the proportion of patients who experienced TEAEs was high (93.5%), less than a third of TEAEs were drug-related and most drug-related TEAEs were mild or moderate in intensity. The results from this study, combined with those from the previously published PK and safety study (12), plus drug exposure matching and extrapolation of efficacy and safety findings from numerous adult studies, contributed to the determination of the recommended clinical pediatric dosing regimen and market authorization of isavuconazole in children 1 year and older for the treatment of IA and IM in the United States.

Successful responses in the pediatric population with proven/probable IA have been shown previously to vary widely based on the antifungal treatment received (15). Additionally, the lack of pediatric-specific standardized definitions for treatment response, plus the relatively small number of pediatric patients in this study compared with those in adult studies, complicates comparisons with historical data (15). Notably, successful response rates at EOT were higher than those of adult patients receiving isavuconazole for proven, probable, or possible invasive mold infections caused by *Aspergillus* spp. (or other filamentous fungi) in the SECURE trial (35.0%) (10). The rate of all-cause case fatality through day 42 was also lower in this study compared with the SECURE trial (20.0%) (10); however, a formal direct comparison of these findings is not possible as the patient populations (children versus adults), the sample sizes, and study design (open-label, non-comparative versus double-blind, and randomized) were different.

Although the majority of patients experienced a TEAE in this study, the high rate and nature of the events—most commonly pyrexia, diarrhea, and vomiting—likely reflect the complex medical histories and underlying diseases often present and are common in those at risk for IFD (1). This is supported by the high proportions of patients in this study who had underlying malignancies or recently resolved/ongoing neutropenia at baseline. Furthermore, the TEAEs and SAEs reported here were broadly similar in nature and frequency to those reported in adults with IFDs in the SECURE study (10). The frequency of these TEAEs did not notably increase during our study. Of particular interest, only a single drug-related SAE was reported in our study (injection site reaction), which resulted in discontinuation with rapid recovery.

Regarding isavuconazole drug exposures resulting from the study dosing regimen, median AUC_ss_ values were lowest in the 1 to <6 years age group versus both the ≥6 to <12 years and ≥12 to <18 years age groups; additionally, more patients in the 1 to <6 years (33.3%) versus ≥6 to <12 years (20.7%) and ≥12 to <18 years (10.3%) cohorts fell below the predefined lower limit of the target exposure threshold (AUC ≥60 mg·h/L) (9, 12). Additionally, no patient achieved drug exposures reaching the pre-defined maximum drug exposure target (AUC_24_: 233 mg·h/L); the lower end of drug exposure was observed in a study in which supra-therapeutic doses of 600 mg isavuconazole (equivalent to 1,116 mg of isavuconazonium sulfate) were used to explore the risk of QT prolongation (14). Higher proportions of younger children aged ≥1 to <3 may fail to achieve desired levels at the studied dose compared to adults. These findings are of particular interest, as the median AUC was lowest in the 12 to <18 years age group in the preceding phase 1 trial of pediatric patients at risk of IFDs (12). The higher variability in the lower age group, small number of patients, and no specific underlying factors other than age, required further thought into the appropriateness of the “one-size fits all” dose for pediatric patients. Therefore, additional analyses were performed to estimate a dose that would be predicted to result in exposures above the lower end of the target exposure threshold in more patients aged 1 to less than 3 years of age; these data demonstrated that an elevated dose of isavuconazole (15 mg/kg isavuconazonium sulfate) may be better suited for this vulnerable group and provide more confidence when using isavuconazole in this age group.

Pediatric clinical trials in rare diseases, such as IFDs, still pose significant challenges, such as a lack of definitive outcome measures and assessment tools, small sample sizes, and a requirement for additional safeguards in vulnerable populations (16, 17). Despite the limited number of patients in this study, the data well characterizes the pharmacokinetic profile for isavuconazole across age groups and demonstrates its safety at the doses and treatment durations expected in the real world. The practice of extrapolating adult clinical outcome data to pediatric patients is supported under the assumption that IA and IM disease pathogenesis and treatment outcomes are similar between adults and children (17). This assumption was considered to be acceptable by the health authorities, which allowed for extrapolation of adult clinical outcome data when considering approval of isavuconazole for pediatric patients. This, when reasonable, can reduce the time to pediatric approval and improve access to life-saving medications for pediatric patients (16).

A strength of this study was that all patients treated with isavuconazole had a diagnosis of at least possible IFD that would require immediate initiation of systemic mold-active antifungal treatment. As such, this study attempted to reflect real-world scenarios as closely as possible. However, there were also several limitations in this study. The sample size available for the pediatric population was small, and further interpretation of outcome rates needs to be conducted with caution; particularly concerning extrapolation of data from smaller sub-populations (e.g., patients aged ≤3 years). The study protocol also relied upon the EORTC/MSG 2008 criteria for definition of IFDs (13), which acknowledge the need to further establish criteria for IFD specific to children as the clinical and radiological manifestations of IFD in patients aged <18 years may differ significantly from those in adults. In addition, the spectrum of underlying conditions leading to immunosuppression differs, radiological characteristics are less specific, and data to support non-culture-based fungal biomarkers in children are sparse. Though the EORTC/MSG 2020 (18) criteria are now available, only the EORTC/MSG 2008 criteria were available when the study began. Therefore, the EORTC/MSG 2008 criteria, which do not incorporate PCR results and other modifications in the assessment of infection, were used in this study and could potentially result in an underestimation of the proven/probable IFD patient population. For instance, four patients with possible IFD had a positive single PCR test (bronchoalveolar lavage [*n* = 3], peritoneal fluid [*n* = 1]) for IA. It is possible that had PCR been specified as a diagnostic criterion in this study, an additional confirmatory PCR test would have been obtained for these patients in line with updated EORTC/MSG criteria (18), and some of these patients may have ultimately been diagnosed with probable IFD.

In conclusion, this phase 2 study demonstrated that isavuconazole had a favorable safety profile consistent with previous studies in adults and was well-tolerated in pediatric patients for the treatment of IFD and the intended treatment durations. Clinical outcomes also suggest a low all-cause case fatality rate, but larger comparator studies are required to determine the comparative effectiveness of this agent for IFD in pediatric patients.

## Data Availability

Researchers may request access to anonymized participant-level data, trial-level data, and protocols from Astellas-sponsored clinical trials at https://www.clinicalstudydatarequest.com/. For the Astellas criteria on data sharing, see https://clinicalstudydatarequest.com/Study-Sponsors/Study-Sponsors-Astellas.aspx.

## SUPPLEMENTAL MATERIAL

Supplemental material aac.00484-24-s0001.pdf Supplemental methods, Tables S1 to S7, and Fig. S1.

ASM does not own the copyrights to Supplemental Material that may be linked to, or accessed through, an article. The authors have granted ASM a non-exclusive, world-wide license to publish the Supplemental Material files. Please contact the corresponding author directly for reuse.
