# Supplementary material for: Safety, outcomes, and pharmacokinetics of isavuconazole as a treatment for invasive fungal diseases in pediatric patients: a non-comparative phase 2 trial
Source: Antimicrob Agents Chemother. 2024 Nov 14;68(12):e00484-24. doi: 10.1128/aac.00484-24 (PMC11642194; doi:10.1128/aac.00484-24)
Supplement: Supplemental material — Supplemental methods, Tables S1 to S7, and Fig. S1. [file aac.00484-24-s0001.pdf]

SUPPLEMENTARY INFORMATION

Supplementary methods

Definition for successful outcome

Investigator guidance for successful clinical outcome was defined as:

Clinical response:

- Resolution of all attributable clinical symptoms and physical findings.
- Partial resolution of attributable clinical symptoms and physical findings.

Mycological response:

- Eradication.
- Presumed eradication.

Radiological response

- Improvement from screening.
- No signs on radiological images at screen (only for proven invasive fungal infections based on other investigations).

Changes in IV dosing regimens by body weight

Patients weighing ≤37 kg: Loading regimen of 10.0 mg/kg infusions every 8 hours (±2) for 6 doses (Days 1 and 2, maximum of 372 mg per individual dose). Maintenance dose of 10.0 mg/kg administered once daily starting 12 to 24 hours after the last loading dose (maximum daily dose of 372 mg per individual dose)

Patients weighing >37 kg: Loading regimen of 372 mg infusions (1 vial) every 8 hours (±2) for 6 doses (Days 1 and 2). Maintenance dose of 372 mg (1 vial) administered once daily starting 12 to 24 hours after the last loading dose.

Changes in oral dosing regimens by body weight

Loading regimen in a capsule form was given every 8 hours (±2 hours) on Days 1 and 2 (a total of six doses), followed by once-daily oral maintenance dosing starting 12 to 24 hours after the last loading dose.

**Body weight**	**Loading/total daily dose***	**Maintenance/total daily dose***
12 to <18 kg	3 × 2 capsules/447 mg	1 × 2 capsules/149 mg
18 to <25 kg	3 × 3 capsules/670.5 mg	1 × 3 capsules/223.5 mg
25 to <32 kg	3 × 4 capsules/894 mg	1 × 4 capsules/298 mg
≥32 kg	3 × 5 capsules/1117.5 mg	1 × 5 capsules/372.5 mg

*Doses are expressed in amounts of isavuconazonium sulfate

Subsequent maintenance doses were administered once daily (24 hours ±2 hours from the previous maintenance dose)

Sampling patterns for safety outcomes

Safety endpoints through to EOT were assessed at each of the following timepoints, by safety outcome:

- AEs: throughout the study, including EOT.
- Vital signs: pre-dose, Days 1, 2, 3, 7, 14, 21, 28, 35, 42, 49, 56, 63, 70, 77, 84 or IA EOT (additionally, for IM patients: Days 85, 115, 145, 180 or EOT).

- ECG: pre-dose, Days 1, 7, 14, 28, 56, 84 or IA EOT (additionally, for IM patients: Days 85, 115, 145, 180 or EOT).
- Laboratory parameters: pre-dose, Day 7, 28, 56, 84, or IA EOT (additionally, for IM patients: Days 115, 145, 180 or EOT).

AE, adverse event; ECG, electrocardiogram; EOT, end of treatment; IA, invasive aspergillosis; IM, invasive mucormycosis; IV, intravenous.

Supplementary tables

Supplementary Table S1: Isavuconazonium sulfate dose changes by body weight as used in the clinical trial

	Body weight	Loading dose† (every 8±2 h over 48 hours)	Maintenance dose (every 24±2 h)‡
IV	≤37 kg	3 x 10 mg/kg	10 mg/kg
	>37 kg	3 x 372 mg	372 mg
Oral*	12 to <18 kg	3 x [149 mg (2 capsules)]	149 mg (2 capsules)
	18 to <25 kg	3 x [223.5 mg (3 capsules)]	223.5 mg (3 capsules)
	25 to <32 kg	3 x [298 mg (4 capsules)]	298 mg (4 capsules)
	≥32 kg	3 x [372.5 mg (5 capsules)]	372.5 mg (5 capsules)

*Oral dose calculated to provide approximately 10 mg/kg dose isavuconazonium sulfate; IV and oral doses are equivalent on a mg/kg basis. In this clinical trial, 74.5 mg isavuconazonium sulfate capsules (equivalent to 40 mg of isavuconazole) were used.

†Loading doses were given every 8 hours (± 2 hours) for a total of 6 doses over 48 hours. The mg dose specified for the loading dose represents the total amount administered in each individual dose given every 8 hours.

‡Maintenance dose was initiated 12 to 24 hours after the final loading dose.

Supplementary Table S2: Treatment and follow-up patient disposition (SAF)

**Parameter**<br>**Category**	**Total (N=31)**
Treatment Status	
Completed	19 (61.3)
Reached Maximum Treatment Duration	12 (38.7)
Successful Outcome	7 (22.6)
Discontinued	12 (38.7)
Adverse Event	3 (9.7)
Death	0
Lack of Efficacy	4 (12.9)
Lost to Follow-Up	0
Protocol Deviation	0
Withdrawal by Participant	0
Withdrawal by Parent/Guardian	0
Other	5 (16.1)
30-Day follow-up status	
Completed	28 (90.3)
Discontinued	3 (9.7)
Adverse Event	0
Death	3 (9.7)
Lost to Follow-Up	0
Withdrawal by Participant	0
Withdrawal by Parent/Guardian	0
Other	0
30-Day follow-up status	
Completed	28 (90.3)

Discontinued	3 (9.7)
Adverse Event	0
Death	3 (9.7)
Lost to Follow-Up	0
Withdrawal by Participant	0
Withdrawal by Parent/Guardian	0
Other	0

SAF, safety analysis set.

Supplementary Table S3: Patient-level overview of discontinuations and drug-related adverse events (SAF)

Patient No.*	Age/sex/race/weight	Treatment duration (days)	Day of discontinuation	Reason for discontinuing treatment	Onset day for drug-related AEs	End day for drug-related AEs	Serious (reason)†/ severity/ course of Event	Drug-related AE
1	1 Years/ Female/ White/ 9.0 kg	95	-	-	-	-	-	-
2	2 Years/ Female/ White/ 12.1 kg	85	-	-	-	-	-	-
3	15 Years/ Female/ White/ 53.1 kg	38	38	OTHER (Worsening of clinical condition related to comorbidities)	-	-	-	-
4	14 Years/ Female/ White/ 74.0 kg	181	-	-	-	-	-	-
5	8 Years/ Female/ White/ 45.5 kg	85	-	-	-	-	-	-

6	17 Years/ Male/ White/ 44.5 kg	181		-	15	181	No/ Moderate/ Intermittent	Gastrointestinal disorders/ Nausea/ mild nausea associated with oral isavuconazole
					68	68	No/ Mild/ Single Episode	Gastrointestinal disorders/ Vomiting/ nonbloody, nonbilious emesis
7	2 Years/ Female/ Asian/ 12.0 kg	84	-	-	-	-	-	-
8	13 Years/ Male/ Other/ 32.2 kg	2	2	ADVERSE EVENT	1	1	Yes (OMI)/ Moderate/ Single Episode	General disorders and administration site conditions/ Infusion site pain/ pain at IV site
					1	1	Yes (OMI)/ Moderate/ Single Episode	General disorders and administration site conditions/ Infusion site pruritus/ localized itching at IV site

					1	1	Yes (OMI)/ Moderate/ Single Episode	Injury, poisoning and procedural complications/Infusion related reaction/ infusion reaction
					2	2	Yes (OMI)/ Moderate/ Single Episode	General disorders and administration site conditions/ Injection site reaction/ injection site reaction
9	6 Years/ Female/ White/ 28.9 kg	28	-	-	-	-	-	-
10	11 Years/ Female/ White/ 28.8 kg	6	6	OTHER (Study not meant for the fungus presumably identified. High risk of failure)	-	-	-	-
11	11 Years/ Female/ Asian/ 42.6 kg	61	-	-	-	-	-	-

12	14 Years/ Male/ White/ 50.5 kg	15	16	LACK OF EFFICACY	9	33	No/ Mild/ Intermittent	Investigations/ Blood bilirubin increased/ Increased Total bilirubin, serum
13	11 Years/ Female/ White/ 47.5 kg	71	73	LACK OF EFFICACY	3	9	No/ Mild/ Continuous	Metabolism and nutrition disorders/ Hypoalbumina emia/ Worsening hypoalbumine mia
14	10 Years/ Female/ Black or African American/ 45.1 kg	47	-	-	-	-	-	-
15	13 Years/ Female/ Other/ 41.3 kg	5	6	OTHER (Subject with 'possible' IFI eligible for enrollment; diagnostic tests to the EORTC/MSG criteria completed within 10 calendar days negative for IA/IM.	-	-	-	-

				Received on 16Feb2022.)				
16	5 Years/ Female/ Other/ 20.3 kg	45	-	-	-	-	-	-
17	16 Years/ Female/ /42.8 kg	16	16	ADVERSE EVENT	-	-	-	-
18	14 Years/ Female/ /63.8 kg	84	-	-	10	19	No/ Mild/ Intermittent	Gastrointestinal disorders/ Nausea/ Nausea
					20	70	No/ Moderate/ Intermittent	Gastrointestinal disorders/ Nausea/ Nausea

	22	36	No/ Mild/ Intermittent	Investigations/ Alanine aminotransfera se increased/ increased alanine aminotransfera se (ALT)
	22	36	No/ Mild/ Continuous	Investigations/ Aspartate aminotransfera se increased /increased aspartate aminotransfera se (AST)
	36	71	No/ Moderate/ Continuous	Investigations/ Alanine aminotransfera se increased/ increased alanine aminotransfera se (ALT)
	36	71	No/ Moderate/ Continuous	Investigations/ Aspartate aminotransfera se increased /increased aspartate aminotransfera

								se<br>(AST)
					71	86	No/<br>Mild/<br>Intermittent	Gastrointestinal disorders/<br>Nausea/<br>Nausea
					71	101	No/<br>Mild/<br>Continuous	Investigations/<br>Alanine aminotransferase increased/<br>increased alanine aminotransferase<br>(ALT)
					71	101	No/<br>Mild/<br>Continuous	Investigations/<br>Aspartate aminotransferase increased<br>/increased aspartate aminotransferase<br>(AST)
					87	113	No/<br>Moderate/<br>Intermittent	Gastrointestinal disorders/<br>Nausea/<br>Nausea
19	1 Years/<br>Female/<br>Other/<br>9.6 kg	99	-	-	-	-	-	-

20	9 Years/ Female/ Asian/ 29.0 kg	84	-	-	-	-	-	-
21	7 Years/ Male/ Asian/ 26.5 kg	84	-	-	-	-	-	-
22	8 Years/ Male/ White/ 43.0 kg	7	7	OTHER (withdrawal by investigator based on very low possability of IA and judged as no further benefit of treatment for patient)	-	-	-	-
23	7 Years/ Female/ White/ 19.2 kg	84	-	-	-	-	-	-
24	13 Years/ Female/ White/ 48.9 kg	67	-	-	-	-	-	-
25	6 Years/ Female/ Asian/ 17.8 kg	84	-	-	-	-	-	-

26	16 Years/ Female/ White/ 55.0 kg	6	7	ADVERSE EVENT	1	Ongoing	No/ Severe/ Intermittent	Vascular disorders/ Hypotension/ hypotension on Isavuconazonium sulfate
27	6 Years/ Female/ White/ 24.4 kg	51		-	-	-	-	-
28	10 Years/ Female/ White/ 60.1 kg	6	6	OTHER (Fungal Infection diagnosis excluded)	5	14	No/ Mild/ Single Episode	Hepatobiliary disorders/ Hypertransaminasaemia/ Hypertransaminemia
					5	14	No/ Mild/ Continuous	Hepatobiliary disorders/ Liver disorder/ Liver Disease
29	3 Years/ Female/ White/ 13.5 kg	22	22	LACK OF EFFICACY	-	-	-	-
30	16 Years/ Female/ White/ 84.3 kg	10	11	LACK OF EFFICACY	2	2	No/ Mild/ Single Episode	Injury, poisoning and procedural complications/ Vascular access site pain/ Loss of venous

								access due to pain during infusion
					2	2	No/ Mild/ Single Episode	General disorders and administration site conditions/ Infusion site pain/ Stinging when administering medication (via infusion)
31	17 Years/ Male/ White/ 44.4 kg	55	-	-	8	43	No/ Mild/ Continuous	Investigations/ Gamma-glutamyl transferase increased/ GGT increased

AE, adverse event; ALT, alanine aminotransferase; AST, aspartate aminotransferase; GGT, gamma-glutamyl transferase; IA, invasive aspergillosis; IFD, invasive fungal disease; IM, invasive mucormycosis; IV, intravenous; NBNB, nausea but no vomiting; OMI, other medical importance; PO, per os; SAF, safety analysis set; TEAE, treatment-emergent adverse event.

*Anonymized patient identification number.

[†]AEs either were identified as serious by the Investigator or upgraded by the sponsor based on the review of the Sponsor's list of ‘always serious’ terms for AEs. Reason for seriousness: D = Death, RPH = Requires or prolongs hospitalization, CA = Congenital anomaly, LT = Life-threatening, PSDI = Persistent or significant disability/incapacity, OMI = Other medical importance, AST = upgraded according to Sponsor's list of ‘always serious’ terms for AEs.

Supplementary Table S4: Causes of death, and impact of discontinuation (SAF)

**Patient No.** *	**Cause of death**	**Date of death from study initiation**	**Last day of study drug dosing**	**Reason for discontinuation**†	**Period from discontinuation to death, days**
1	Cardiovascular collapse	Day 61	Day 38	Worsening of clinical condition related to comorbidities	23
2	Septic shock	Day 26	Day 6	Hypotension (severe TEAE)	20
3	Progressive IFD	Day 15	Day 10	Lack of efficacy	5

IFD, invasive fungal disease; SAF, safety analysis set; TEAE, treatment-emergent adverse event.

*Anonymized patient identification number.

†All deaths were preceded by study discontinuation.

Supplementary Table S5. Drug-related TEAEs and deaths through EOT (SAF)

System Organ Class<br>Preferred Term<br>(MedDRA v23.0)	Subgroup: Proven/probable IA (n=12)		Proven/probable IM (n=1)		Possible IFD*† (n=16)		Other IFD‡ (n=2)		Total (N=31)	
	n (%)	#Events	n (%)	#Events	n (%)	#Events	n (%)	#Events	n (%)	#Events
Overall, n (%)	3 (25.0)	16	1 (100.0)	1	5 (31.3)	7	0	0	9 (29.0)	24
Gastrointestinal disorders	1 (8.3)	4	0	0	1 (6.3)	2	0	0	2 (6.5)	6
Nausea	1 (8.3)	4	0	0	1 (6.3)	1	0	0	2 (6.5)	5
Vomiting	0	0	0	0	1 (6.3)	1	0	0	1 (3.2)	1
General disorders and administration site conditions	2 (16.7)	4	0	0	0	0	0	0	2 (6.5)	4
Infusion site pain	2 (16.7)	2	0	0	0	0	0	0	2 (6.5)	2
Infusion site pruritus	1 (8.3)	1	0	0	0	0	0	0	1 (3.2)	1
Injection site reaction	1 (8.3)	1	0	0	0	0	0	0	1 (3.2)	1‡
Hepatobiliary disorders	0	0	0	0	1 (6.3)	2	0	0	1 (3.2)	2
Hypertransaminasaemia	0	0	0	0	1 (6.3)	1	0	0	1 (3.2)	1

Liver disorder	0	0	0	0	1 (6.3)	1	0	0	1 (3.2)	1
Injury, poisoning and procedural complications	2 (16.7)	2	0	0	0	0	0	0	2 (6.5)	2
Infusion related reaction	1 (8.3)	1	0	0	0	0	0	0	1 (3.2)	1
Vascular access site pain	1 (8.3)	1	0	0	0	0	0	0	1 (3.2)	1
Investigations	1 (8.3)	6	1 (100.0)	1	1 (6.3)	1	0	0	3 (9.7)	8
Alanine aminotransferase increased	1 (8.3)	3	0	0	0	0	0	0	1 (3.2)	3
Aspartate aminotransferase increased	1 (8.3)	3	0	0	0	0	0	0	1 (3.2)	3
Blood bilirubin increased	0	0	1 (100.0)	1	0	0	0	0	1 (3.2)	1
Gamma-glutamyltransferase increased	0	0	0	0	1 (6.3)	1	0	0	1 (3.2)	1
Metabolism and nutrition disorders	0	0	0	0	1 (6.3)	1	0	0	1 (3.2)	1
Hypoalbuminaemia	0	0	0	0	1 (6.3)	1	0	0	1 (3.2)	1
Vascular disorders	0	0	0	0	1 (6.3)	1	0	0	1 (3.2)	1

Hypotension	0	0	0	0	1 (6.3)	1	0	0	1 (3.2)	1[§]

EORTC/MSG, European Organisation for Research and Treatment of Cancer/Invasive Fungal Infections Cooperative Group and Mycoses Study Group; IA, invasive aspergillosis; IFD, invasive fungal disease; IM, invasive mucormycosis; MedDRA, Medical Dictionary for Regulatory Activities; SAF, safety analysis set; TEAE, treatment-emergent adverse event.

*Two of the patients with possible IFD were subsequently determined by the independent adjudication committee as not meeting the EORTC/MSG 2008 criteria (13) for possible IFD.

[†]An investigator assessment of IFD diagnosis was used; diagnostic tests to assess whether the disease was ‘proven’ or ‘probable’ IA or IM, according to the EORTC/MSG 2008 criteria (13), were completed within 10 days after the first dose of the study drug.

[‡]Other IFDs were defined as IFDs assessed not to be IA or IM.

[§]Drug-related TEAEs that led to discontinuation.

Supplementary Table S6: Common non-serious TEAEs experienced in >5% of patients (SAF)

System Organ Class Preferred Term (MedDRA v23.0)	Subgroup: Proven/probable IA (n=12)		Proven/probable IM (n=1)		Possible IFD*† (n=16)		Other IFD‡ (n=2)		Total (N=31)	
	n (%)	#Events	n (%)	#Events	n (%)	#Events	n (%)	#Events	n (%)	#Events
Overall‡	10 (83.3)	159	1 (100.0)	12	15 (93.8)	182	2 (100.0)	20	28 (90.3)	373
Overall of ≥ 5% threshold events§	10 (83.3)	159	1 (100.0)	12	15 (93.8)	182	2 (100.0)	20	28 (90.3)	373
Blood and lymphatic system disorders	4 (33.3)	9	0	0	5 (31.3)	13	1 (50.0)	1	10 (32.3)	23
Anaemia	2 (16.7)	2	0	0	1 (6.3)	4	0	0	3 (9.7)	6
Lymphopenia	1 (8.3)	1	0	0	1 (6.3)	1	1 (50.0)	1	3 (9.7)	3
Thrombocytopenia	1 (8.3)	1	0	0	2 (12.5)	2	0	0	3 (9.7)	3
Neutropenia	1 (8.3)	1	0	0	1 (6.3)	3	0	0	2 (6.5)	4
Cardiac disorders	1 (8.3)	2	0	0	4 (25.0)	6	0	0	5 (16.1)	8
Tachycardia	1 (8.3)	1	0	0	2 (12.5)	2	0	0	3 (9.7)	3
Ear and labyrinth disorders	2 (16.7)	3	0	0	0	0	0	0	2 (6.5)	3
Ear pain	2 (16.7)	3	0	0	0	0	0	0	2 (6.5)	3
Gastrointestinal disorders	8 (66.7)	41	1 (100.0)	1	10 (62.5)	20	2 (100.0)	4	21 (67.7)	66
Diarrhoea	4 (33.3)	5	1 (100.0)	1	3 (18.8)	3	0	0	8 (25.8)	9
Vomiting	4 (33.3)	11	0	0	3 (18.8)	3	0	0	7 (22.6)	14
Stomatitis	2 (16.7)	3	0	0	2 (12.5)	2	1 (50.0)	1	5 (16.1)	6
Nausea	2 (16.7)	6	0	0	2 (12.5)	2	0	0	4 (12.9)	8
Aphthous ulcer	3 (25.0)	6	0	0	1 (6.3)	1	0	0	4 (12.9)	7

Abdominal distension	2 (16.7)	2	0	0	1 (6.3)	1	0	0	3 (9.7)	3
Abdominal pain	0	0	0	0	2 (12.5)	2	1 (50.0)	1	3 (9.7)	3
Constipation	2 (16.7)	2	0	0	0	0	1 (50.0)	1	3 (9.7)	3
General disorders and administration site conditions	7 (58.3)	16	1 (100.0)	2	7 (43.8)	12	2 (100.0)	4	17 (54.8)	34
Pyrexia	3 (25.0)	7	0	0	5 (31.3)	5	1 (50.0)	1	9 (29.0)	13
Non-cardiac chest pain	2 (16.7)	2	0	0	2 (12.5)	2	1 (50.0)	1	5 (16.1)	5
Pain	0	0	1 (100.0)	2	0	0	1 (50.0)	1	2 (6.5)	3
Chills	1 (8.3)	1	0	0	1 (6.3)	1	0	0	2 (6.5)	2
Immune system disorders	2 (16.7)	3	0	0	1 (6.3)	1	0	0	3 (9.7)	4
Drug hypersensitivity	2 (16.7)	3	0	0	0	0	0	0	2 (6.5)	3
Infections and infestations	8 (66.7)	14	0	0	6 (37.5)	15	0	0	14 (45.2)	29
Rhinitis	1 (8.3)	2	0	0	2 (12.5)	2	0	0	3 (9.7)	4
Folliculitis	2 (16.7)	2	0	0	0	0	0	0	2 (6.5)	2
Oral herpes	2 (16.7)	2	0	0	0	0	0	0	2 (6.5)	2
COVID-19	1 (8.3)	1	0	0	1 (6.3)	1	0	0	2 (6.5)	2
Investigations	4 (33.3)	14	1 (100.0)	1	7 (43.8)	23	1 (50.0)	1	13 (41.9)	39
QRS axis abnormal	0	0	0	0	2 (12.5)	4	0	0	2 (6.5)	4
Transaminases increased	1 (8.3)	1	0	0	1 (6.3)	1	0	0	2 (6.5)	2
Metabolism and nutrition disorders	3 (25.0)	6	1 (100.0)	3	8 (50.0)	22	0	0	12 (38.7)	31
Hypokalaemia	1 (8.3)	1	1 (100.0)	2	2 (12.5)	4	0	0	4 (12.9)	7
Hypoalbuminaemia	0	0	0	0	4 (25.0)	4	0	0	4 (12.9)	4

Hyperkalaemia	1 (8.3)	1	0	0	1 (6.3)	3	0	0	2 (6.5)	4
Hypophosphataemia	0	0	0	0	2 (12.5)	2	0	0	2 (6.5)	2
Musculoskeletal and connective tissue disorders	4 (33.3)	8	0	0	3 (18.8)	4	1 (50.0)	2	8 (25.8)	14
Pain in extremity	1 (8.3)	3	0	0	0	0	1 (50.0)	1	2 (6.5)	4
Arthralgia	1 (8.3)	1	0	0	1 (6.3)	1	0	0	2 (6.5)	2
Muscle spasms	2 (16.7)	2	0	0	0	0	0	0	2 (6.5)	2
Nervous system disorders	2 (16.7)	2	1 (100.0)	1	2 (12.5)	2	1 (50.0)	1	6 (19.4)	6
Headache	2 (16.7)	2	0	0	1 (6.3)	1	0	0	3 (9.7)	3
Renal and urinary disorders	1 (8.3)	1	1 (100.0)	1	2 (12.5)	5	1 (50.0)	1	5 (16.1)	8
Dysuria	0	0	1 (100.0)	1	0	0	1 (50.0)	1	2 (6.5)	2
Respiratory, thoracic and mediastinal disorders	7 (58.3)	16	1 (100.0)	2	7 (43.8)	24	2 (100.0)	2	17 (54.8)	44
Epistaxis	1 (8.3)	1	0	0	2 (12.5)	3	0	0	3 (9.7)	4
Cough	0	0	0	0	3 (18.8)	3	0	0	3 (9.7)	3
Respiratory distress	2 (16.7)	2	0	0	1 (6.3)	1	0	0	3 (9.7)	3
Rhinorrhoea	2 (16.7)	2	0	0	1 (6.3)	1	0	0	3 (9.7)	3
Tachypnoea	1 (8.3)	1	0	0	1 (6.3)	1	1 (50.0)	1	3 (9.7)	3
Oropharyngeal pain	2 (16.7)	3	0	0	0	0	0	0	2 (6.5)	3
Dyspnoea	1 (8.3)	1	1 (100.0)	1	0	0	0	0	2 (6.5)	2
Hypoxia	0	0	0	0	1 (6.3)	1	1 (50.0)	1	2 (6.5)	2
Pulmonary oedema	0	0	0	0	2 (12.5)	2	0	0	2 (6.5)	2
Lung opacity	1 (8.3)	1	0	0	1 (6.3)	1	0	0	2 (6.5)	2

Skin and subcutaneous tissue disorders	5 (41.7)	16	0	0	6 (37.5)	6	0	0	11 (35.5)	22
Dry skin	3 (25.0)	3	0	0	0	0	0	0	3 (9.7)	3
Pruritus	3 (25.0)	3	0	0	0	0	0	0	3 (9.7)	3
Urticaria	2 (16.7)	3	0	0	0	0	0	0	2 (6.5)	3
Petechiae	2 (16.7)	2	0	0	0	0	0	0	2 (6.5)	2
Rash	1 (8.3)	1	0	0	1 (6.3)	1	0	0	2 (6.5)	2
Vascular disorders	2 (16.7)	2	1 (100.0)	1	3 (18.8)	8	0	0	6 (19.4)	11
Hypertension	0	0	1 (100.0)	1	2 (12.5)	2	0	0	3 (9.7)	3
Hypotension	0	0	0	0	3 (18.8)	3	0	0	3 (9.7)	3
Pallor	2 (16.7)	2	0	0	0	0	0	0	2 (6.5)	2

EORTC/MSG, European Organisation for Research and Treatment of Cancer/Invasive Fungal Infections Cooperative Group and Mycoses Study Group; IA, invasive aspergillosis; IFD, invasive fungal disease; IM, invasive mucormycosis; MedDRA, Medical Dictionary for Regulatory Activities; SAF, safety analysis set; TEAE, treatment-emergent adverse event.

*Two of the patients with possible IFD were subsequently determined by the independent adjudication committee as not meeting the EORTC/MSG 2008 criteria (13) for possible IFD.

†An investigator assessment of IFD diagnosis was used; diagnostic tests to assess whether the disease was ‘proven’ or ‘probable’ IA or IM, according to the EORTC/MSG 2008 criteria (13), were completed within 10 days after the first dose of the study drug.

‡Other IFDs were defined as IFDs assessed not to be IA or IM.

Supplementary Table S7: Overview of serious TEAEs (SAF)

System Organ Class<br>Preferred Term<br>(MedDRA v23.0)	Subgroup: Proven/probable IA (n=12)		Proven/probable IM (n=1)		Possible IFD*† (n=16)		Other IFD‡ (n=2)		Total (N=31)	
	n (%)	#Events	n (%)	#Events	n (%)	#Events	n (%)	#Events	n (%)	#Events
Overall	9 (75.0)	22	0	0	9 (56.3)	20	0	0	18 (58.1)	42
Blood and Lymphatic System Disorders	2 (16.7)	3	0	0	0	0	0	0	2 (6.5)	3
Febrile neutropenia	2 (16.7)	3	0	0	0	0	0	0	2 (6.5)	3
Cardiac Disorders	1 (8.3)	1	0	0	1 (6.3)	1	0	0	2 (6.5)	2
Cardio-respiratory arrest	1 (8.3)	1	0	0	0	0	0	0	1 (3.2)	1
Pericardial effusion	0	0	0	0	1 (6.3)	1	0	0	1 (3.2)	1
Ear and Labyrinth Disorders	1 (8.3)	1	0	0	0	0	0	0	1 (3.2)	1
Ear pain	1 (8.3)	1	0	0	0	0	0	0	1 (3.2)	1
Gastrointestinal Disorders	2 (16.7)	4	0	0	1 (6.3)	1	0	0	3 (9.7)	5
Stomatitis	2 (16.7)	4	0	0	0	0	0	0	2 (6.5)	4
Abdominal distension	0	0	0	0	1 (6.3)	1	0	0	1 (3.2)	1
General Disorders and Administration Site Conditions	1 (8.3)	3	0	0	0	0	0	0	1 (3.2)	3
Infusion site pain	1 (8.3)	1	0	0	0	0	0	0	1 (3.2)	1
Infusion site pruritus	1 (8.3)	1	0	0	0	0	0	0	1 (3.2)	1
Injection site reaction	1 (8.3)	1	0	0	0	0	0	0	1 (3.2)	1
Hepatobiliary Disorders	0	0	0	0	1 (6.3)	1	0	0	1 (3.2)	1

Venoocclusive liver disease	0	0	0	0	1 (6.3)	1	0	0	1 (3.2)	1
Immune System Disorders	0	0	0	0	1 (6.3)	1	0	0	1 (3.2)	1
Graft versus host disease	0	0	0	0	1 (6.3)	1	0	0	1 (3.2)	1
Infections and Infestations	2 (16.7)	2	0	0	8 (50.0)	12	0	0	10 (32.3)	14
Septic shock	0	0	0	0	3 (18.8)	4	0	0	3 (9.7)	4
Bacteraemia	0	0	0	0	1 (6.3)	1	0	0	1 (3.2)	1
Bacterial sepsis	1 (8.3)	1	0	0	0	0	0	0	1 (3.2)	1
Brain abscess	0	0	0	0	1 (6.3)	1	0	0	1 (3.2)	1
Escherichia sepsis	0	0	0	0	1 (6.3)	1	0	0	1 (3.2)	1
Pneumococcal sepsis	0	0	0	0	1 (6.3)	1	0	0	1 (3.2)	1
Pneumonia pseudomonal	0	0	0	0	1 (6.3)	1	0	0	1 (3.2)	1
Pneumonia	1 (8.3)	1	0	0	0	0	0	0	1 (3.2)	1
Sepsis	0	0	0	0	1 (6.3)	1	0	0	1 (3.2)	1
Streptococcal sepsis	0	0	0	0	1 (6.3)	1	0	0	1 (3.2)	1
Vascular device infection	0	0	0	0	1 (6.3)	1	0	0	1 (3.2)	1
Injury, Poisoning and Procedural Complications	1 (8.3)	1	0	0	0	0	0	0	1 (3.2)	1
Infusion related reaction	1 (8.3)	1	0	0	0	0	0	0	1 (3.2)	1
Musculoskeletal and Connective Tissue Disorders	2 (16.7)	3	0	0	1 (6.3)	1	0	0	3 (9.7)	4
Arthralgia	1 (8.3)	2	0	0	0	0	0	0	1 (3.2)	2
Rhabdomyolysis	0	0	0	0	1 (6.3)	1	0	0	1 (3.2)	1
Synovitis	1 (8.3)	1	0	0	0	0	0	0	1 (3.2)	1

Renal and Urinary Disorders	0	0	0	0	1 (6.3)	1	0	0	1 (3.2)	1
Anuria	0	0	0	0	1 (6.3)	1	0	0	1 (3.2)	1
Respiratory, Thoracic and Mediastinal Disorders	2 (16.7)	2	0	0	1 (6.3)	1	0	0	3 (9.7)	3
Haemoptysis	1 (8.3)	1	0	0	0	0	0	0	1 (3.2)	1
Pleural effusion	0	0	0	0	1 (6.3)	1	0	0	1 (3.2)	1
Respiratory failure	1 (8.3)	1	0	0	0	0	0	0	1 (3.2)	1
Social Circumstances	1 (8.3)	1	0	0	0	0	0	0	1 (3.2)	1
Social problem	1 (8.3)	1	0	0	0	0	0	0	1 (3.2)	1
Vascular Disorders	1 (8.3)	1	0	0	1 (6.3)	1	0	0	2 (6.5)	2
Circulatory collapse	1 (8.3)	1	0	0	0	0	0	0	1 (3.2)	1
Hypertension	0	0	0	0	1 (6.3)	1	0	0	1 (3.2)	1

EORTC/MSG, European Organisation for Research and Treatment of Cancer/Invasive Fungal Infections Cooperative Group and Mycoses Study Group; IA, invasive aspergillosis; IFD, invasive fungal disease; IM, invasive mucormycosis; FAS, full analysis set; MedDRA, Medical Dictionary for Regulatory Activities; SAF, safety analysis set; TEAE, treatment-emergent adverse event.

*Two of the patients with possible IFD were subsequently determined by the independent adjudication committee as not meeting the EORTC/MSG 2008 criteria (13) for possible IFD.

†An investigator assessment of IFD diagnosis was used; diagnostic tests to assess whether the disease was ‘proven’ or ‘probable’ IA or IM, according to the EORTC/MSG 2008 criteria (13), were completed within 10 days after the first dose of the study drug.

‡Other IFDs were defined as IFDs assessed not to be IA or IM.

Supplementary figures

Supplemental Figure S1. Patient baseline IFD disposition

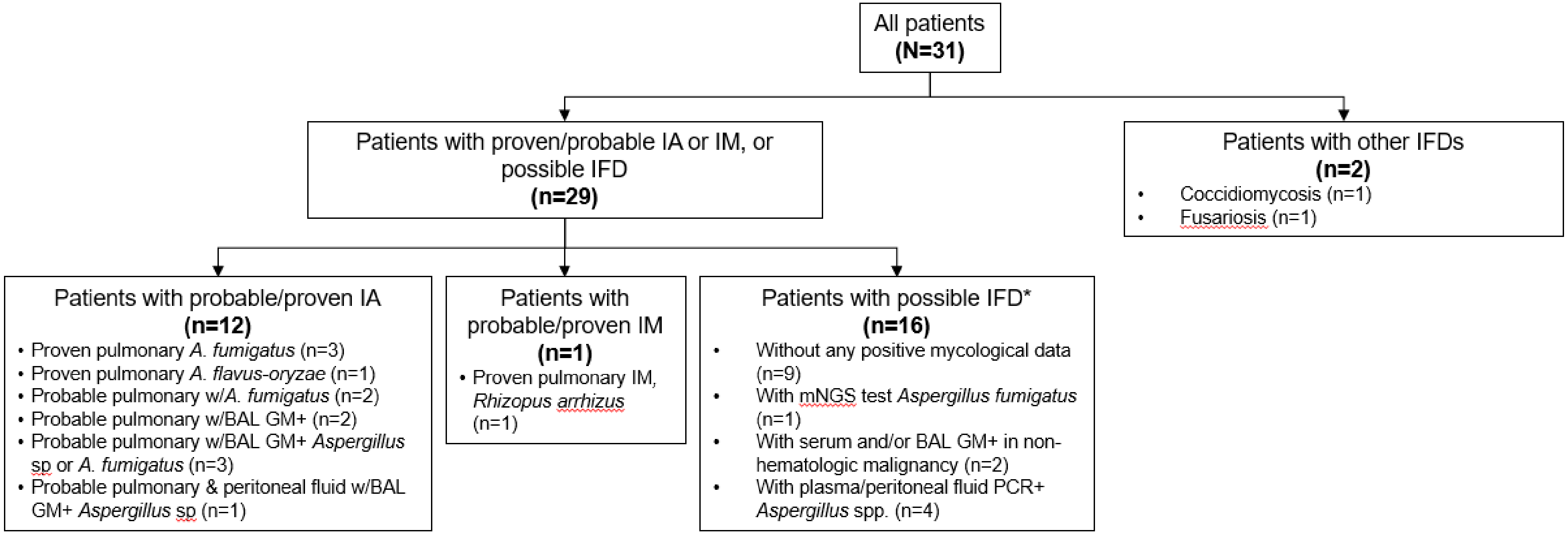

IA, invasive aspergillus; BAL GM+, positive galactomannan testing of bronchoalveolar lavage fluid; Galactomannan IFD, invasive fungal disease; IM, invasive mucormycosis; mNGS, metagenomic next-generation sequencing of plasma; PCR, polymerase chain reaction.

*One patient had an underlying diagnosis of systemic lupus erythematosus, while the second patient had a history of multi-visceral transplants and was PCR-positive for *Aspergillus fumigatus* at enrollment.
